# Average Volume of Alcohol Consumed, Drinking Patterns, and Metabolic Syndrome in Older Korean Adults

**DOI:** 10.2188/jea.JE20120065

**Published:** 2013-03-05

**Authors:** Min-Ho Shin, Sun-Seog Kweon, Jin-Su Choi, Jung-Ae Rhee, Hae-Sung Nam, Seul-Ki Jeong, Kyeong-Soo Park, So-Yeon Ryu, Seong-Woo Choi, Bok-Hee Kim, Young-Hoon Lee

**Affiliations:** 1 Department of Preventive Medicine, Chonnam National University Medical School, Gwangju, South Korea; 2 Jeonnam Regional Cancer Center, Chonnam National University Hwasun Hospital, Hwasun, South Korea; 3 Department of Preventive Medicine, Chungnam National University College of Medicine, Daejeon, South Korea; 4 Department of Neurology, Chonbuk National University Medical School and Hospital, Jeonju, Jeonbuk, South Korea; 5 Department of Preventive Medicine, Seonam University College of Medicine, Namwon, Jeonbuk, South Korea; 6 Department of Preventive Medicine, Chosun University Medical School, Gwangju, South Korea; 7 Department of Food & Nutrition, Chosun University College of Natural Sciences, Gwangju, South Korea; 8 Department of Preventive Medicine, Wonkwang University College of Medicine, Iksan, Jeonbuk, South Korea; 9 Institute of Wonkwang Medical Science, Iksan, Jeonbuk, South Korea

**Keywords:** metabolic syndrome, alcohol consumption, cross-sectional study

## Abstract

**Background:**

Controversial results have been reported on the relationship between alcohol intake and metabolic syndrome (MetS). We examined the association of average volume of alcohol consumed and drinking patterns with MetS and its components.

**Methods:**

This study was conducted as a baseline survey for the Dong-gu Study of adults aged 50 years or older. Drinking patterns were assessed using a structured interview, and average volume of alcohol consumed was calculated. MetS was defined according to the updated version of the National Cholesterol Education Program.

**Results:**

Compared with individuals who never drank, the adjusted odds ratio (OR) for the prevalence of MetS was significantly higher in men who consumed 2.1 to 4.0 drinks/day (OR, 1.53; 95% CI, 1.17–2.00) and greater than 4.0 drinks/day (OR, 1.63; 95% CI, 1.23–2.14), whereas no significant association was observed in women. Significant dose-response relationships between average volume of alcohol consumed and all metabolic components were observed in men. A usual quantity of 5 to 6 drinks/drinking day (OR, 1.57; 95% CI, 1.19–2.09), 7 or more drinks/drinking day (OR, 1.88; 95% CI, 1.45–2.44), and binge drinking on at least 1 occasion/week (OR, 1.33; 95% CI, 1.01–1.76) were associated with a significantly higher OR for prevalence of MetS in men; however, none of these drinking patterns were associated with MetS in women.

**Conclusions:**

Unhealthy drinking patterns such as high usual quantity and binge drinking were significantly associated with MetS, suggesting that the effect of alcohol consumption on MetS should be considered in the context of drinking pattern, particularly in men.

## INTRODUCTION

Numerous epidemiologic studies have described a J-shaped or U-shaped curve for the relationship between alcohol consumption and the risk of cardiovascular disease morbidity and mortality.^1^^–^^3^ Cumulative scientific evidence has demonstrated that light to moderate alcohol drinking is associated with cardiovascular protective effects, whereas heavy alcohol intake results in poor health outcomes.^3^^–^^7^ The biologic mechanisms for the beneficial effects of light to moderate alcohol consumption on the development of coronary artery disease may include increased high-density lipoprotein cholesterol (HDL-C),^8^ increased fibrinolysis and decreased platelet aggregation,^5^^,^^9^ reduced inflammation,^10^^,^^11^ and increased insulin sensitivity.^12^^,^^13^

Metabolic syndrome (MetS) is a constellation of metabolic risk factors, including central adiposity, high blood pressure, dyslipidemia, and hyperglycemia. Epidemiologic studies have shown that MetS is a strong indicator for the increased risk of cardiovascular morbidity and mortality.^14^^–^^16^ Although it is well established that light to moderate alcohol consumption is associated with a reduced risk for cardiovascular morbidity and mortality, epidemiologic evidence for an association between alcohol consumption and MetS is inconsistent and controversial.^17^^–^^30^ Furthermore, because most studies of the relationship between alcohol consumption and MetS have focused on average volume of alcohol consumption, the association between MetS and drinking patterns (eg, drinking frequency, usual quantity consumed, and frequency of binge drinking) is unclear.^31^^–^^33^

Thus, we investigated the association of average volume of alcohol consumed with MetS and its components among community-dwelling adults aged 50 years or older. In addition, we evaluated the effect of drinking patterns, such as drinking frequency, usual quantity consumed, and frequency of binge drinking, on MetS and its components.

## METHODS

### Study subjects

This study was conducted as a baseline survey within the framework of the Dong-gu Study, which is an ongoing prospective population-based cohort study that was designed to investigate the prevalence, incidence, and risk factors for chronic disease in an urban population. National resident registration records were used to identify potential participants. From 2007 to 2010, 34 040 eligible subjects who were aged 50 years or older and resided in the Dong-gu district of Gwangju Metropolitan City in South Korea were invited to participate by mail and telephone. A total of 9260 subjects were enrolled (response rate: 27.2%; 3711 men and 5549 women).

A total of 848 with a self-reported history of cerebrovascular disease or coronary heart disease were excluded from the analysis. Ultimately, 8181 subjects (3178 men and 5003 women) were included in the analyses after excluding an additional 192 subjects who did not provide information on their alcohol consumption and 39 subjects who did not provide information on anthropometric or metabolic measures. This study was conducted in accordance with the Declaration of Helsinki guidelines. All subjects were fully informed of the study content and gave written informed consent for the use of their data. The present study was approved by the institutional review board of Chonnam National University Hospital.

### Alcohol consumption

The current drinking status of each subject was assessed using the following 2 questions: “Before the date of this study, have you ever drunk any alcoholic beverages?” and “In the last 12 months have you drunk any alcoholic beverages?”. Participants who answered “no” to both questions were classified as never drinkers (lifetime abstainers). Participants who answered “yes” to the first question and “no” to the second question were classified as former drinkers. Current drinkers were defined as participants who answered “yes” to both questions. Current drinkers were asked 2 related questions: “On a day when you drink alcohol, how many drinks do you usually have (usual quantity of alcohol consumed)?” and “How often do you have a drink containing alcohol, per month (drinking frequency)?”. The average volume of alcohol consumed per day (drinks/day) was calculated from the average number of alcoholic beverages consumed by current drinkers. The number of standard drinks of each beverage type on a single occasion was assessed. We used the additional questions to differentiate former drinkers (those who had stopped drinking for health or other reasons) from lifetime abstainers (never drinkers). On the basis of the average volume of alcohol consumed, we classified subjects as never drinkers (lifetime abstainers), former drinkers, and current drinkers. Current drinkers were further classified (consumption of ≤1.0, 1.1–2.0, 2.1–4.0, or >4.0 drinks/day of alcohol for men and ≤0.5, 0.6–1.0, 1.1–2.0, and >2.0 drinks/day of alcohol for women).^34^

Drinking frequency was classified as less than 1 day/week, 1 to 2 days/week, and 3 or more days/week. Usual quantity of alcohol consumed was classified as 1 to 2 drinks/drinking day, 3 to 4 drinks/drinking day, 5 to 6 drinks/drinking day, and 7 or more drinks/drinking day for men and as 1 to 2 drinks/drinking day, 3 to 4 drinks/drinking day, and 5 or more drinks/drinking day for women. All subjects who currently drank were also asked about their frequency of binge drinking, which was defined as the consumption, on a single occasion, of 7 or more standard drinks for men and 5 or more drinks for women. Current drinkers were classified into 3 categories according to binge drinking frequency: none, low binge drinkers (<1 occasion/week), and high binge drinkers (≥1 occasion/week).

### Measurement of metabolic components

During the period 2007–2010, all participants underwent a yearly standardized physical examination conducted by experienced research staff in the Gwangju Dong-gu health center between April and July. Anthropometric measurements were made in light clothing and without shoes. Body mass index was calculated as weight (kg) divided by height squared (m^2^). Waist circumference was measured to the nearest 0.1 cm at the midpoint between the lower border of the rib cage and the upper hip bone during expiration. Blood pressure was measured with an appropriate size cuff on the right upper arm using a standard mercury sphygmomanometer (Baumanometer; WA Baum Co., Inc., Copiague, NY, USA) after at least 5 min of rest in a sitting position. Three consecutive systolic and diastolic blood pressure readings, which were determined to the nearest 2 mm Hg, were recorded at an interval of 1 min, and the average was used for the analysis.

Blood samples were collected from an antecubital vein of each subject after a 12-h overnight fast. Serum was separated from the samples on-site and stored at −70°C until analysis, which occurred within 4 weeks. Serum total cholesterol, HDL-C, triglycerides (TG), and fasting blood glucose (FBG) levels were measured using enzymatic techniques. Low-density lipoprotein cholesterol (LDL-C) was calculated by the Friedewald formula. Aspartate aminotransferase (AST) and γ-glutamyl transpeptidase (GGT) activity were measured using commercial reagent kits (Daiichi Pure Chemicals, Tokyo, Japan). All samples were analyzed using an automatic analyzer (Model 7600 chemical analyzer; Hitachi Ltd., Tokyo, Japan).

### Definition of MetS

MetS was defined by the presence of 3 or more of the following 5 conditions: abdominal obesity (ABO; waist circumference ≥90 cm for men and ≥85 cm for women), high triglycerides (HTG; triglycerides ≥150 mg/dL), low HDL-C (LHDLC; HDL-C <40 mg/dL for men and <50 mg/dL for women), high BP (HBP; SBP ≥130 mm Hg and/or DBP ≥85 mm Hg and/or current use of antihypertensive medication), and high FBG (HFBG; FBG ≥100 mg/dL and/or current use of hypoglycemic agents).^35^ We defined ABO using the Korean Society for the Study of Obesity criteria.^36^ We applied a new cut-off point for impaired FBG, as defined by the American Diabetes Association.^37^

### Covariates

Information on age, education level, marital status, healthcare service, cigarette smoking, exercise, self-reported medical history, and medication use of each subject was assessed by research staff, using a standardized questionnaire. Education level was classified as at least 6 years, 7–12 years, and 13 years or more. Marital status was dichotomized into married or other (unmarried, divorced, separated, or widowed). Healthcare service was categorized as National Health Insurance or Medical Aid. Smoking status was classified into never smoker, former smoker, and current smoker. Exercise was categorized as none, irregular, and regular based on the frequency of recreational activity and exercise during a week.

### Statistical analysis

All analyses were performed separately by sex because of the difference in amounts of alcohol consumed, and because of the significant interaction between sex and alcohol consumption with MetS. Demographic, anthropometric, and metabolic variables were expressed as mean ± SD or as a ratio based on the sex-specific alcohol consumption categories. Analysis of variance and the chi-square test were used to identify statistical differences in continuous and categorical variables, respectively. After adjusting for age, smoking status, exercise, educational level, marital status, and healthcare service, odds ratios (ORs) and 95% CIs of sex-specific alcohol consumption categories for MetS and its components were calculated using multiple logistic regression analysis and compared with never drinkers (reference). After excluding former drinkers, the alcohol consumption categories were treated as a continuous term in the multiple logistic regression analysis to test for a linear trend in the relationship between alcohol intake and MetS and metabolic components. In addition, ORs of the sex-specific alcohol consumption categories for MetS were calculated to compare with current nondrinkers (never and former drinkers).

After excluding never and former drinkers, ORs were calculated and compared to the reference groups in order to examine the association between drinking patterns and MetS in 3 models. Model 1 was unadjusted, model 2 was adjusted for age, smoking status, exercise, educational level, marital status, and healthcare services, and model 3 was further adjusted for usual quantity consumed in the analysis for drinking frequency, for drinking frequency in the analysis for usual quantity, and for average volume of alcohol consumption in the analysis for binge drinking frequency. Because usual quantity and frequency may reflect different aspects of alcohol drinking, we hypothesized that drinking frequency would be significantly associated with MetS, independent of usual quantity. Thus, to investigate the independent association between drinking frequency and MetS, usual quantity was further controlled in model 3. Equally, we examined the independent association between usual quantity and MetS, while further controlling for frequency in model 3. Additionally, we hypothesized that binge drinking would be significantly associated with MetS independent of the average volume of alcohol consumed.

A *P* value of less than 0.05 was considered statistically significant. All statistical analyses were performed using PASW statistics 18 (SPSS Inc., Chicago, IL, USA).

## RESULTS

### Characteristics of study population

Demographic, anthropometric, and metabolic variables according to alcohol consumption and sex are shown in Table 1. Among the study population, 69.2% of men and 31.2% of women were current alcohol drinkers, whereas 18.7% of men and 62.7% of women were never drinkers.

**Table 1.** Demographic, anthropometric, and metabolic characteristics of participants, according to alcohol consumption

	Men (*n* = 3178)			Women (*n* = 5003)		
	
	Never drinkers (*n* = 594)	Former drinkers (*n* = 384)	Current drinkers (*n* = 2200)	Never drinkers (*n* = 3137)	Former drinkers (*n* = 306)	Current drinkers (*n* = 1560)
Age, yr	67.8 ± 8.3	68.0 ± 8.3	65.1 ± 7.8^a,b^	65.3 ± 8.1	65.6 ± 8.5	61.8 ± 7.7^a,b^
BMI, kg/m^2^	23.4 ± 3.1	23.6 ± 2.9	24.0 ± 2.7^a,b^	24.6 ± 3.1	24.5 ± 3.0	24.7 ± 2.8
WC, cm	85.8 ± 8.8	86.7 ± 8.2	87.3 ± 7.4^a^	88.5 ± 9.2	88.3 ± 9.3	88.4 ± 8.4
SBP, mm Hg	123.2 ± 16.8	123.0 ± 16.6	125.3 ± 16.4^a,b^	122.6 ± 16.7	123.0 ± 17.4	121.2 ± 17.0^a^
DBP, mm Hg	74.2 ± 10.3	73.8 ± 10.4	76.3 ± 10.6^a,b^	73.4 ± 9.8	73.5 ± 9.8	74.0 ± 10.2
FBG, mg/dL	108.9 ± 23.2	111.9 ± 25.6	114.3 ± 26.9^a^	106.6 ± 23.4	107.6 ± 24.3	106.3 ± 22.0
TC, mg/dL	194.0 ± 38.6	185.6 ± 36.3^a^	192.8 ± 37.2^b^	209.4 ± 39.5	209.1 ± 39.6	209.5 ± 38.6
HDL-C, mg/dL	47.5 ± 11.1	45.9 ± 10.5	51.2 ± 12.4^a,b^	52.6 ± 11.7	50.8 ± 11.3^a^	54.4 ± 12.1^a,b^
LDL-C, mg/dL	120.4 ± 33.2	113.6 ± 31.5^a^	113.0 ± 33.3^a^	129.8 ± 35.8	128.7 ± 36.5	128.4 ± 34.3
TG, mg/dL	136.2 ± 91.7	133.4 ± 84.6	154.6 ± 126.7^a,b^	139.8 ± 86.1	151.7 ± 98.0	137.6 ± 101.8
Smoking status, %						
Never	39.2	19.3^a^	23.1^a,b^	97.5	93.2^a^	94.7^a^
Former	40.8	57.8	49.3	1.1	4.2	2.6
Current	20.0	22.9	27.6	1.4	2.6	2.7
Exercise, %						
None	48.4	48.3^a^	42.7^a,b^	58.2	58.8^a^	54.1^a,b^
Irregular	37.1	31.1	40.0	31.2	25.2	33.5
Regular	14.5	20.6	17.3	10.6	16.0	12.4
Education level, %						
≤6 yr	27.2	29.3	24.9	53.6	63.4^a^	48.8^a,b^
7–12 yr	46.5	43.2	49.0	39.7	32.4	45.3
≥13 yr	26.3	27.5	26.1	6.7	4.2	5.9
Marital status, %						
Married	90.0	87.2	91.5^b^	69.8	63.5^a^	71.9^b^
Others^c^	10.0	12.8	8.5	30.2	36.5	28.1
Healthcare service, %						
NHI	92.9	89.1	94.8^b^	91.7	88.6	93.9^a,b^
Medical Aid	7.1	10.9	5.2	8.3	11.4	6.1

Data are expressed as means ± SD or as percentages.

BMI, body mass index; WC, waist circumference; SBP, systolic blood pressure; DBP, diastolic blood pressure; FBG, fasting blood glucose; TC, total cholesterol; HDL-C, high-density lipoprotein cholesterol; LDL-C, low-density lipoprotein cholesterol; TG, triglycerides; NHI, National Health Insurance.

^a^*P* < 0.05, compared with never drinkers.

^b^*P* < 0.05, compared with former drinkers.

^c^Unmarried, divorced, separated, widowed.

### Relationship between alcohol consumption and MetS

MetS was present in 45.1% of the study population (38.3% of men and 49.5% of women). The prevalence and ORs for MetS associated with average volume of alcohol consumed are presented in Table 2 for men and Table 3 for women. There was a graded positive association between average volume of alcohol consumed and prevalence of MetS in men but not in women (*P*-trend <0.001 and 0.131, respectively). After adjusting for age, smoking status, exercise, educational level, marital status, and healthcare service, a significant dose-response relationship between average volume of alcohol consumed and MetS was observed in men but not in women (*P*-trend <0.001 and 0.376, respectively). Compared with never drinkers, the ORs for MetS were significantly higher in men who consumed 2.1 to 4.0 drinks/day (OR, 1.53; 95% CI, 1.17–2.00) and greater than 4.0 drinks/day (OR, 1.63; 95% CI, 1.23–2.14), whereas ORs did not significantly differ in women.

**Table 2.** Relationship of average volume of alcohol consumption with metabolic syndrome and its components in men

	Never drinkers (*n* = 594)	Former drinkers (*n* = 384)	Current drinkers (*n* = 2200)				*P*-trend^c^

			≤1.0 drinks/day (*n* = 1038)	1.1–2.0 drinks/day (*n* = 391)	2.1–4.0 drinks/day (*n* = 403)	>4.0 drinks/day (*n* = 368)	
Prevalence, %							
Metabolic syndrome	34.3	40.4	35.5	37.9	43.7	45.4	<0.001
Metabolic components							
HBP	53.0	50.8	53.6	56.0	61.8	61.7	0.001
HFBG	66.0	68.8	69.6	74.2	77.4	78.5	<0.001
LHDLC	23.7	30.7	19.6	16.1	9.7	12.2	<0.001
HTG	29.8	30.4	31.9	35.3	42.4	44.8	<0.001
ABO	32.2	37.0	32.4	34.3	40.7	40.2	0.001
OR (95% CI)^a^							
Metabolic syndrome	1.00	1.30 (0.99–1.71)	1.08 (0.87–1.34)	1.24 (0.94–1.62)	1.53 (1.17–2.00)	1.63 (1.23–2.14)	<0.001
Metabolic components							
HBP	1.00	0.91 (0.70–1.19)	1.08 (0.87–1.33)	1.29 (0.99–1.69)	1.72 (1.31–2.25)	1.81 (1.37–2.40)	<0.001
HFBG	1.00	1.13 (0.85–1.49)	1.16 (0.93–1.45)	1.48 (1.11–1.98)	1.77 (1.32–2.39)	1.92 (1.40–2.62)	<0.001
LHDLC	1.00	1.47 (1.10–1.97)	0.83 (0.65–1.07)	0.65 (0.47–0.91)	0.35 (0.24–0.51)	0.42 (0.29–0.61)	<0.001
HTG	1.00	0.99 (0.74–1.32)	1.07 (0.85–1.34)	1.21 (0.91–1.60)	1.46 (1.11–1.93)	1.49 (1.13–1.98)	0.001
ABO	1.00	1.28 (0.97–1.68)	1.03 (0.82–1.28)	1.20 (0.91–1.59)	1.55 (1.18–2.04)	1.61 (1.21–2.13)	<0.001
OR (95% CI)^b^							
Metabolic syndrome	1.00		0.97 (0.80–1.17)	1.11 (0.87–1.42)	1.37 (1.08–1.75)	1.46 (1.14–1.88)	0.001

HBP, high blood pressure; HFBG, high fasting blood glucose; LHDLC, low high-density lipoprotein cholesterol; HTG, high triglycerides; ABO, abdominal obesity; OR, odds ratio.

^a^Compared with never drinkers after adjustment for age, smoking status, exercise, educational level, marital status, and healthcare service.

^b^Compared with current nondrinkers (never + former) after adjustment for age, smoking status, exercise, educational level, marital status, and healthcare service.

^c^*P* for trend was obtained by chi-square test (prevalence) or logistic regression (OR) using categories of alcohol consumption as a continuous variable after excluding former drinkers.

**Table 3.** Relationship of average volume of alcohol consumption with metabolic syndrome and its components in women

	Never drinkers (*n* = 3137)	Former drinkers (*n* = 306)	Current drinkers (*n* = 1560)				*P*-trend^c^

			≤0.5 drinks/day (*n* = 1326)	0.6–1.0 drinks/day (*n* = 104)	1.1–2.0 drinks/day (*n* = 80)	>2.0 drinks/day (*n* = 50)	
Prevalence, %							
Metabolic syndrome	50.0	53.9	47.8	44.2	46.3	50.0	0.131
Metabolic components							
HBP	52.4	52.3	47.4	51.0	53.8	50.0	0.026
HFBG	56.3	58.2	54.1	64.4	58.8	68.0	0.691
LHDLC	43.4	50.3	39.4	32.7	21.3	18.0	<0.001
HTG	33.0	39.2	31.5	29.8	28.8	36.0	0.310
ABO	66.0	67.6	67.1	60.6	66.3	72.0	0.611
OR (95% CI)^a^							
Metabolic syndrome	1.00	1.13 (0.89–1.45)	1.08 (0.94–1.23)	0.98 (0.65–1.48)	1.01 (0.64–1.61)	1.15 (0.64–2.06)	0.376
Metabolic components							
HBP	1.00	0.95 (0.74–1.22)	0.99 (0.86–1.13)	1.32 (0.88–1.99)	1.27 (0.80–2.03)	1.19 (0.66–2.14)	0.355
HFBG	1.00	1.04 (0.82–1.33)	1.01 (0.88–1.15)	1.57 (1.04–2.39)	1.22 (0.77–1.94)	1.66 (0.90–3.05)	0.074
LHDLC	1.00	1.25 (0.99–1.59)	0.88 (0.77–1.01)	0.65 (0.43–0.99)	0.35 (0.21–0.61)	0.26 (0.13–0.55)	<0.001
HTG	1.00	1.29 (1.01–1.65)	0.98 (0.85–1.13)	0.93 (0.60–1.43)	0.81 (0.49–1.33)	1.15 (0.64–2.08)	0.638
ABO	1.00	1.06 (0.82–1.37)	1.19 (1.03–1.37)	0.94 (0.62–1.41)	1.20 (0.74–1.96)	1.38 (0.73–2.61)	0.032
OR (95% CI)^b^							
Metabolic syndrome	1.00		1.07 (0.93–1.22)	0.97 (0.64–1.46)	1.00 (0.63–1.59)	1.13 (0.63–2.03)	0.451

HBP, high blood pressure; HFBG, high fasting blood glucose; LHDLC, low high-density lipoprotein cholesterol; HTG, high triglycerides; ABO, abdominal obesity; OR, odds ratio.

^a^Compared with never drinkers after adjustment for age, smoking status, exercise, educational level, marital status, and healthcare service.

^b^Compared with current nondrinkers (never + former) after adjustment for age, smoking status, exercise, educational level, marital status, and healthcare service.

^c^*P* for trend was obtained by chi-square test (prevalence) or logistic regression (OR) using categories of alcohol consumption as a continuous variable after excluding former drinkers.

Significant dose-response relationships between alcohol consumption and all metabolic components were observed in men; however, LHDLC was the only component that showed a dose-response relationship in women. Among metabolic components, the ORs for HBP, HTG, and ABO were significantly higher in men who consumed 2.1 to 4.0 drinks/day and greater than 4.0 drinks/day, and the OR for HFBG was significantly higher in men who consumed 1.1 to 2.0 drinks/day, 2.1 to 4.0 drinks/day, and greater than 4.0 drinks/day, as compared with never drinkers. The ORs for LHDLC were significantly lower in men who consumed 1.1 to 2.0 drinks/day, 2.1 to 4.0 drinks/day, and greater than 4.0 drinks/day. The ORs for ABO and HFBG were significantly higher in women who consumed 0.5 drinks/day or less and 0.6 to 1.0 drinks/day, respectively. The ORs for LHDLC were significantly lower in women who consumed 0.6 to 1.0 drinks/day, 1.1 to 2.0 drinks/day, and greater than 2.0 drinks/day.

### Comparison of the relationship between alcohol drinking and MetS

Data on the association between MetS and light drinking (≤2.0 drinks/day for men and ≤1.0 drinks/day for women) and heavy drinking (>2.0 drinks/day for men and >1.0 drinks/day for women) are shown in Table 4. The OR for MetS associated with heavy drinking was significantly higher than that associated with light drinking among men (OR, 1.39; 95% CI, 1.15–1.68) but not women (OR, 0.96; 95% CI, 0.66–1.41). Compared with light drinkers, significantly higher ORs for HBP, HFBG, HTG, and ABO, and a significantly lower OR for LHDLC, were found in male heavy drinkers, whereas a significantly lower OR was found only for LHDLC in female heavy drinkers.

**Table 4.** Prevalence and odds ratios of metabolic syndrome and its components among current light and heavy drinkers

	Men (*n* = 2200)			Women (*n* = 1560)		
	
	Light (≤2.0 drinks/day) (*n* = 1429)	Heavy (>2.0 drinks/day) (*n* = 771)	*P*-value	Light (≤1.0 drinks/day) (*n* = 1430)	Heavy (>1.0 drinks/day) (*n* = 130)	*P*-value
Prevalence, %						
Metabolic syndrome	36.1	44.5	<0.001	47.6	47.7	0.976
Metabolic components						
HBP	54.2	61.7	0.001	47.7	52.3	0.313
HFBG	70.8	78.0	<0.001	54.9	62.3	0.103
LHDLC	18.6	10.9	<0.001	38.9	20.0	<0.001
HTG	32.8	43.6	<0.001	31.4	31.5	0.974
ABO	32.9	40.5	<0.001	66.6	68.5	0.673
OR (95% CI)^a^						
Metabolic syndrome	1.00	1.39 (1.15–1.68)	0.001	1.00	0.96 (0.66–1.41)	0.963
Metabolic components						
HBP	1.00	1.53 (1.27–1.85)	<0.001	1.00	1.19 (0.81–1.75)	0.364
HFBG	1.00	1.45 (1.17–1.79)	0.001	1.00	1.28 (0.87–1.88)	0.206
LHDLC	1.00	0.50 (0.38–0.65)	<0.001	1.00	0.37 (0.23–0.58)	<0.001
HTG	1.00	1.31 (1.09–1.59)	0.005	1.00	0.91 (0.61–1.35)	0.637
ABO	1.00	1.47 (1.22–1.79)	<0.001	1.00	1.11 (0.74–1.66)	0.616

HBP, high blood pressure; HFBG, high fasting blood glucose; LHDLC, low high-density lipoprotein cholesterol; HTG, high triglycerides; ABO, abdominal obesity; OR, odds ratio.

^a^Adjusted for age, smoking status, exercise, educational level, marital status, and healthcare service.

### Relationship between drinking patterns and MetS

The relationship between drinking pattern (eg, drinking frequency, usual quantity, and frequency of binge drinking) and MetS was examined among current drinkers (Tables 5 and 6). In model 3, a high usual quantity (5–6 drinks/drinking day and ≥7 drinks/drinking day) and frequent binge drinking (≥1 occasion/week) were significantly associated with a higher adjusted OR for MetS in men (OR, 1.57; 95% CI, 1.19–2.09 for 5–6 drinks/drinking day; OR, 1.88; 95% CI, 1.45–2.44 for ≥7 drinks/drinking day, and OR, 1.33; 95% CI, 1.01–1.76 for ≥1 occasion/week binge drinkers, respectively), whereas drinking frequency was not associated with MetS. In contrast, none of the drinking patterns were significantly associated with MetS in women.

**Table 5.** Relationship between drinking patterns and metabolic syndrome among current drinkers

	Model 1^a^	Model 2^b^	Model 3^c,d^
Men			
Drinking frequency^a,b,c^			
<1 day/week (*n* = 602)	1.00	1.00	1.00
1–2 days/week (*n* = 757)	0.99 (0.79–1.24)	1.00 (0.80–1.25)	0.86 (0.68–1.09)
≥3 days/week (*n* = 841)	1.17 (0.94–1.15)	1.15 (0.92–1.43)	0.95 (0.76–1.20)
Usual quantity^a,b,d^			
1–2 drinks/drinking day (*n* = 585)	1.00	1.00	1.00
3–4 drinks/drinking day (*n* = 513)	1.11 (0.86–1.42)	1.15 (0.89–1.48)	1.17 (0.90–1.51)
5–6 drinks/drinking day (*n* = 404)	1.47 (1.13–1.91)	1.53 (1.17–2.01)	1.57 (1.19–2.09)
≥7 drinks/drinking day (*n* = 698)	1.71 (1.36–2.15)	1.83 (1.43–2.34)	1.88 (1.45–2.44)
Women			
Drinking frequency^a,b,c^			
<1 day/week (*n* = 1188)	1.00	1.00	1.00
1–2 days/week (*n* = 244)	1.04 (0.79–1.37)	1.13 (0.85–1.52)	1.09 (0.81–1.46)
≥3 days/week (*n* = 128)	1.05 (0.73–1.51)	0.89 (0.60–1.31)	0.83 (0.56–1.24)
Usual quantity^a,b,d^			
1–2 drinks/drinking day (*n* = 1121)	1.00	1.00	1.00
3–4 drinks/drinking day (*n* = 301)	1.04 (0.81–1.35)	1.09 (0.83–1.43)	1.09 (0.83–1.43)
≥5 drinks/drinking day (*n* = 138)	1.12 (0.79–1.60)	1.32 (0.91–1.92)	1.35 (0.91–1.99)

Data are presented as odds ratio (95% CI).

HBP, high blood pressure; HFBG, high fasting blood glucose; LHDLC, low high-density lipoprotein cholesterol; HTG, high triglycerides; ABO, abdominal obesity.

^a^Unadjusted.

^b^Adjusted for age, smoking status, exercise, educational level, marital status, and healthcare service.

^c^Adjusted for age, smoking status, exercise, educational level, marital status, healthcare service, and usual quantity.

^d^Adjusted for age, smoking status, exercise, educational level, marital status, healthcare service, and drinking frequency.

**Table 6.** Relationship between binge drinking and metabolic syndrome among current drinkers

	Model 1^a^	Model 2^b^	Model 3^c^
Men			
None (*n* = 644)	1.00	1.00	1.00
<1 occasion/week (*n* = 711)	1.11 (0.89–1.39)	1.13 (0.90–1.41)	1.10 (0.87–1.38)
≥1 occasion/week (*n* = 830)	1.51 (1.22–1.86)	1.51 (1.21–1.89)	1.33 (1.01–1.76)
Women			
None (*n* = 1091)	1.00	1.00	1.00
<1 occasion/week (*n* = 371)	1.06 (0.84–1.34)	1.23 (0.96–1.58)	1.28 (0.98–1.66)
≥1 occasion/week (*n* = 93)	0.96 (0.63–1.47)	1.16 (0.74–1.83)	1.35 (0.77–2.40)

Data are presented as odds ratio (95% CI).

HBP, high blood pressure; HFBG, high fasting blood glucose; LHDLC, low high-density lipoprotein cholesterol; HTG, high triglycerides; ABO, abdominal obesity.

Binge drinking is defined as the consumption of ≥7 drinks for men and ≥5 drinks for women on a single occasion.

Fifteen men and 5 women who did not provide information on their binge drinking were excluded.

^a^Unadjusted.

^b^Adjusted for age, smoking status, exercise, educational level, marital status, and healthcare service.

^c^Adjusted for age, smoking status, exercise, educational level, marital status, healthcare service, and average volume of alcohol consumption.

In men, high drinking frequency was significantly associated with a higher OR for HBP and HFBG and a lower OR for LHDLC. A high usual quantity was significantly associated with a higher OR for HBP, HFBG, HTG, and ABO, and a lower OR for LHDLC. Also, a high frequency of binge drinking was significantly associated with a higher OR for HTG and ABO. In women, high drinking frequency was significantly associated with a lower OR for LHDLC, and high usual quantity was significantly associated with a higher OR for HBP and ABO and a lower OR for LHDLC (Table 7).

**Table 7.** Relationship between drinking patterns and components of metabolic syndrome among current drinkers

	Metabolic components				

	HBP	HFBG	LHDLC	HTG	ABO
Men (*n* = 2200)					
Drinking frequency^a^					
<1 day/week (*n* = 602)	1.00	1.00	1.00	1.00	1.00
1–2 days/week (*n* = 757)	1.12 (0.89–1.41)	1.30 (1.01–1.67)	0.60 (0.45–0.80)	0.93 (0.73–1.18)	1.03 (0.81–1.30)
≥3 days/week (*n* = 841)	1.35 (1.07–1.70)	1.35 (1.05–1.74)	0.44 (0.32–0.60)	1.15 (0.91–1.47)	1.04 (0.82–1.32)
Usual quantity^b^					
1–2 drinks/day (*n* = 585)	1.00	1.00	1.00	1.00	1.00
3–4 drinks/day (*n* = 513)	1.11 (0.86–1.42)	1.05 (0.81–1.38)	0.70 (0.51–0.97)	1.22 (0.93–1.59)	1.22 (0.93–1.59)
5–6 drinks/day (*n* = 404)	1.43 (1.08–1.89)	1.41 (1.03–1.92)	0.77 (0.53–1.12)	1.36 (1.01–1.82)	1.69 (1.26–2.26)
≥7 drinks/day (*n* = 698)	1.55 (1.21–2.02)	1.45 (1.09–1.92)	0.70 (0.50–0.98)	1.32 (1.01–1.73)	2.04 (1.56–2.67)
Binge drinking frequency^c^					
None (*n* = 644)	1.00	1.00	1.00	1.00	1.00
<1 day/week (*n* = 711)	1.10 (0.88–1.38)	1.22 (0.96–1.57)	0.90 (0.67–1.20)	1.11 (0.87–1.41)	1.24 (0.98–1.57)
≥1 day/week (*n* = 830)	1.21 (0.91–1.59)	1.14 (0.84–1.55)	0.83 (0.57–1.21)	1.43 (1.07–1.91)	1.47 (1.10–1.96)
Women (*n* = 1560)					
Drinking frequency^a^					
<1 day/week (*n* = 1188)	1.00	1.00	1.00	1.00	1.00
1–2 days/week (*n* = 244)	1.19 (0.88–1.60)	1.53 (1.13–2.07)	0.57 (0.42–0.79)	0.90 (0.65–1.23)	1.01 (0.74–1.38)
≥3 days/week (*n* = 128)	1.02 (0.68–1.52)	1.43 (0.96–2.15)	0.49 (0.32–0.76)	0.95 (0.63–1.44)	0.74 (0.49–1.12)
Usual quantity^b^					
1–2 drinks/day (*n* = 1121)	1.00	1.00	1.00	1.00	1.00
3–4 drinks/day (*n* = 301)	1.45 (1.10–1.90)	1.10 (0.84–1.44)	0.79 (0.60–1.05)	1.10 (0.83–1.46)	1.42 (1.06–1.91)
≥5 drinks/day (*n* = 138)	1.58 (1.07–2.34)	1.25 (0.84–1.85)	0.64 (0.42–0.98)	1.10 (0.73–1.66)	1.68 (1.10–2.58)
Binge drinking frequency^c^					
None (*n* = 1091)	1.00	1.00	1.00	1.00	1.00
<1 day/week (*n* = 371)	1.28 (0.99–1.67)	1.18 (0.91–1.53)	0.85 (0.65–1.10)	1.36 (1.04–1.78)	1.53 (1.15–2.03)
≥1 day/week (*n* = 93)	1.37 (0.77–2.42)	0.94 (0.53–1.67)	0.81 (0.43–1.51)	1.58 (0.87–2.86)	1.12 (0.62–2.02)

Data are presented as odds ratio (95% CI).

HBP, high blood pressure; HFBG, high fasting blood glucose; LHDLC, low high-density lipoprotein cholesterol; HTG, high triglycerides; ABO, abdominal obesity.

^a^Adjusted for age, smoking status, exercise, educational level, marital status, healthcare service, and usual quantity.

^b^Adjusted for age, smoking status, exercise, educational level, marital status, healthcare service, and drinking frequency.

^c^Binge drinking is defined as the consumption of ≥7 drinks for men and ≥5 drinks for women on a single occasion; adjusted for age, smoking status, exercise, educational level, marital status, healthcare service, and average volume of alcohol consumption. Fifteen men and 5 women who did not provide information on their binge drinking were excluded.

## DISCUSSION

We found a significant dose-response relationship—not a J-shaped relationship—between average volume of alcohol consumed and MetS and its components in men, whereas only LHDLC and ABO were associated with MetS in women. Compared with light drinkers, male heavy drinkers but not female heavy drinkers had a significantly higher OR for prevalence of MetS and metabolic components. In men, unhealthy drinking patterns such as higher drinking intensity and binge drinking were significantly associated with an increased OR for prevalence of MetS and its components, whereas, in women, unhealthy drinking patterns were significantly associated with an increased OR for the prevalence of some metabolic components, but not MetS. We found that not only average daily alcohol volume but also drinking patterns was significantly associated with MetS and related components of MetS, especially in men. Although light to moderate alcohol intake has protective effects on cardiovascular disease morbidity and mortality,^5^^–^^7^ findings from epidemiologic studies of the association between alcohol consumption and MetS are controversial. Some studies have found that moderate alcohol consumption is associated with a lower risk of MetS, whereas heavy alcohol drinking increases the risk of MetS (J- or U-shaped relation).^17^^–^^19^ Recently, a systematic literature review of published observational studies showed that alcohol consumption less than 40 g/day in men and less than 20 g/day in women (ie, responsible alcohol intake) significantly reduced MetS risk.^20^ However, no significant association was found between alcohol drinking and MetS in other studies.^21^^–^^23^ In our study, heavy drinking, namely, 2.1 to 4.0 drinks/day or greater than 4.0 drinks/day, was significantly associated with a higher OR for prevalence of MetS, whereas no significant association was found between light drinking (<1.0 drinks/day or 1.1–2.0 drinks/day) and MetS prevalence in men. Compared with never drinkers, mild to moderate alcohol drinking and drinking pattern had no significant beneficial effect on MetS in either sex. This inconsistent relationship across studies might be due to the multifaceted and complex mechanistic relationship between alcohol consumption and each of the MetS components.^24^^–^^30^

Most studies of the relationship of alcohol intake with cardiovascular outcomes or MetS have collected information on average volume of alcohol consumption. Because few epidemiologic studies included information on drinking patterns, the association between drinking patterns and diseases is unclear. The Western New York Health Study of 2818 lifetime drinking adults showed that drinking intensity (drinks per drinking day), but not drinking frequency (drinking days per week), was significantly associated with MetS and all MetS components.^31^ Recently, the 1999–2002 National Health and Nutrition Examination Survey of 1529 current drinkers found that people with unhealthy drinking patterns, such as higher than usual drinking quantity, drinking exceeding dietary guidelines, and frequent binge drinking, were at a significantly increased risk for MetS, even after controlling for drinking frequency.^32^ Significant associations between unhealthy drinking patterns and individual metabolic components were also observed.^32^ Evidence from a meta-analysis showed that drinking patterns modify the effect of alcohol consumption on coronary heart disease (CHD) risk.^33^ Binge and heavy irregular drinking is associated with increased CHD risk, whereas the well-established protective or beneficial effect of alcohol on CHD risk has been confirmed among regular drinkers.^33^

In agreement with previous studies,^31^^–^^33^ we observed that unhealthy drinking patterns, such as higher usual quantity and binge drinking, were significantly associated with an increased OR for the prevalence of MetS and its components among men. Although there was no significant association between drinking patterns and MetS among women, some metabolic components were related to drinking patterns in our study. The LHDLC component of MetS was inversely associated with drinking pattern in both sexes. LHDLC was more strongly associated with drinking frequency than with usual quantity in men. This association was not apparent in women. The beneficial effect of high drinking frequency on HDL cholesterol was diminished by the detrimental effect on other MetS components. In contrast, the potent harmful effect of high usual quantity on abdominal obesity and blood pressure affected the significant association between usual quantity and MetS. Similar associations were observed in women; however, the results were not statistically significant.

It has been suggested that drinking frequency is more highly correlated than usual quantity consumed with average volume of alcohol consumed. In our correlational analysis, we also examined this phenomenon (*r* = 0.794 for drinking frequency and *r* = 0.673 for usual quantity; *P* for difference <0.001). In our study, 30.0% of male binge drinkers (≥1 day/week) and 34.4% of female binge drinkers (≥1 occasion/week) were classified as light drinkers (<2.0 drinks/day for men and <1.0 drink/day for women) when only average volume of alcohol consumed was used. Previous epidemiologic studies using average volume as an indicator of alcohol intake were limited in detecting the effects of usual quantity (drinks per drinking day) or binge drinking. Therefore, it is important to include drinking pattern measures in epidemiologic studies of alcohol-related health problems and to consider drinking patterns when defining moderate drinking.^32^

One methodologic issue in assessing the health impact of alcohol consumption is selecting the reference category. Most studies of the relationship between alcohol intake and MetS combined lifetime abstainers and former drinkers in a single category of current nondrinkers and used this as a reference group.^17^^–^^19^^,^^21^^–^^23^ Thus, the J- or U-shaped association between alcohol consumption and cardiovascular mortality may be the result of including former drinkers in the nondrinking reference group.^6^ Former drinkers are at increased risk for death from cardiovascular disease because they may have given up alcohol drinking due to health problems.^38^ However, limited and mixed information on nondrinkers combined into 1 reference category might conceal the real effects of non-alcohol use and may lead to an artifactual protective effect of alcohol use.^20^^,^^39^ In this study, a crude J-shaped relationship between average volume of alcohol consumed and the OR for prevalence of MetS was found in men when current nondrinkers were used as the reference group, whereas a significant dose-response relationship was observed when only lifetime abstainers were used as the reference group. Additional prospective studies using abstainers as a reference group are needed to confirm the association between alcohol consumption and the risk for MetS.

The absence of an association between alcohol consumption and MetS in women may be explained by the fact that women consume less alcohol than men. Furthermore, in Korean culture, drinking alcohol is less accepted among women than among men; thus under-reporting of alcohol consumption may have been more prevalent in women. These measurement errors may have affected the relationship between alcohol consumption and MetS.

This study had some limitations and considerations. First, we cannot draw any causal inferences from our data because of the cross-sectional design. Second, the study response rate was considerably lower than that reported in other studies. It is possible that the low response rate does not reflect the current status of the entire population. Third, we did not analyze the effect of beverage type on MetS. Therefore, we could not evaluate the effect of different types of alcoholic beverages on the prevalence of MetS and its components. Fourth, other drinking patterns such as drinking with meals and age at first alcohol consumption were not included in the analysis.

Nevertheless, this study had several strengths. First, it included a relatively large sample size as compared with previous studies. Second, we separated former drinkers who had stopped drinking for health problems or other reasons from lifetime abstainers (never drinkers). Analysis using nondrinkers as a reference group might have resulted in biased or weakened results as compared with the results obtained by using lifetime abstainers and former drinkers separately.

In conclusion, there was a significant association between average volume of alcohol consumed and MetS in older men but not women. Unhealthy drinking patterns such as higher usual quantity and binge drinking were significantly associated with MetS in men but not women. Therefore, the effect of alcohol consumption on MetS should be considered in the context of drinking patterns, especially in men. From a public health perspective, we recommend that heavy drinkers and binge drinkers stop or control their unhealthy drinking habits.
